# Visuo-Attentional and Phonological Deficits Explored in French Students with Dyslexia: Eye Movements Recorded during a Phonological Lexical Decision Task

**DOI:** 10.3390/neurolint16020022

**Published:** 2024-03-01

**Authors:** Aikaterini Premeti, Frédéric Isel, Maria Pia Bucci

**Affiliations:** 1 MoDyCo, UMR 7114 CNRS Université Paris Nanterre, 92001 Nanterre, France; fisel@parisnanterre.fr; 2 ICAR UMR 5191, CNRS, ENS Lyon—Site Descartes, University of Lyon 2, 69342 Lyon, France

**Keywords:** developmental dyslexia, phonological lexical decision task, eye movements

## Abstract

Whether dyslexia is caused by phonological or attentional dysfunction remains a widely debated issue. To enrich this debate, we compared the eye movements of 32 French university students with (14 students) and without (18 students) dyslexia while performing a delayed phonological lexical decision task on 300 visually presented stimuli. The processing stimuli involved either a lexical (i.e., words) or a non-lexical route relying on a grapheme-phoneme correspondence (pseudohomophones and pseudowords), while other stimuli involved only a visual search (consonant and symbol sequences). We recorded the number of fixations, the duration of the first fixation and the amplitude of saccades made on the stimuli. Compared to the controls, the participants with dyslexia made more fixations while reading regardless of the type of stimulus (lexical and non-lexical). Crucially, the participants with dyslexia exhibited longer first fixations in particular while reading phonologically challenging stimuli such as pseudohomophones and pseudowords compared to stimuli involving a simple visual search (consonants, symbols). Taken together, these results suggest that both visual and phonological impairments may be implicated in dyslexia, supporting the hypothesis that dyslexia is a multifactorial deficit.

## 1. Introduction

The main cause of dyslexia is considered to be a phonological deficit, i.e., a difficulty in the use of grapheme-to-phoneme conversion rules [1,2,3]. However, many researchers query whether a phonological deficit is the main cause of dyslexia [4,5,6]. Other theories such as auditory deficits [7], working memory impairment [8], attentional abnormalities [9], magnocellular abnormalities [10], and more recently, temporal oscillatory sampling deficit [11] have been proposed as alternative explanations. Among them, the visuo-attentional deficit hypothesis [9] postulates that participants with dyslexia have a reduced visuo-attentional span, i.e., they can treat a smaller number of letters simultaneously within a single fixation, which could explain the abnormal eye movement pattern reported in participants with dyslexia during reading. Irrespective of the language, it was observed that participants with dyslexia make a large number of saccades of small amplitude [12,13,14,15,16,17,18,19], several retro-saccades in order to re-fixate a word [17,20,21,22], and longer fixation durations (for a recent review, see [23]). Interestingly, this abnormal oculomotor pattern was observed in German participants not only when reading a text but also when reading a list of pseudowords [24] or when reading isolated words of different lengths in Italian [25] and in German [26] and words with a different lexical frequency in German [26].

There is an ongoing debate on whether phonological or visuo-attentional deficits play a more prominent role in dyslexia. As highlighted in a recent review by Valdois [27], numerous studies have questioned whether participants with dyslexia struggle only with verbal alphanumeric stimuli (i.e., letters and numbers) that require both visuo-attentional and phonological processing, or whether these difficulties extend to symbol strings that comprise non-verbalized elements such as false fonts, obscure geometric forms, or letters from unknown alphabets, which involve purely visuo-attentional processing. The key idea is that if readers with dyslexia struggle only with alphanumeric stimuli and not with symbols, their deficits can be attributed to phonological issues. On the other hand, if they have difficulties in processing both types of stimuli their deficits are more visuo-attentional in nature, since they are observed independently of whether the stimulus requires purely visuo-attentional processing or phonological processing.

The research findings in this area have been contradictory. Pammer et al. [28] used a symbol identification task to examine children with dyslexia. In this task children were presented with an array of symbols consisting of vertical and horizontal lines. Their objective was to decide which of the two subsequent arrays of symbols, presented after the initial one, was identical to the original array. The researchers found a poorer performance in children with dyslexia as compared to their age-matched controls, concluding that this result could be attributed to visuo-attentional difficulties. Similar results were observed with adults with dyslexia in a study by Jones et al. [29] who used the same task and stimuli as Pammer et al. [28]. Similarly, Zhao et al. [30] found poorer processing among dyslexic Chinese schoolchildren in the upper grades during a symbol identification task of a previously five-figure string. In the same vein, Lobier et al. [31] used both verbal and non-verbal stimuli in a visual categorization task with children with dyslexia and age-matched controls and found that the children with dyslexia were less efficient compared to the control group independently of the stimulus type, supporting a possible underlying visuo-attentional deficit in dyslexia.

Controversially, other studies failed to find differences between participants with and without dyslexia when processing non-verbal stimuli (both in studies with children [32,33,34] and in studies with adults [35,36]). For example, Ziegler et al. [33] found deficits in French children with dyslexia, but these deficits only emerged when alphanumeric stimuli were used and not during symbol processing. This differentiation was evident in the higher error rates observed with alphanumeric stimuli compared to symbols. Analogous results were observed in Chinese children with dyslexia [34], as well as in adults during a task using Georgian letters [36]. These results are in favor of a phonological deficit in dyslexia, since difficulties are present only when dealing with stimuli requiring phonological activation, whereas symbols that require only visuo-attentional processing do not pose the same difficulties.

Another line of research on eye movements has tried to address the question of whether dyslexia has its roots in linguistic and phonological factors or originates more from visuo-attentional aspects. These studies involve the examination of children and adults with dyslexia while performing reading tasks that encompass linguistic information processing as well as non-verbal tasks that involve visual processing only. The core idea of this is that if participants with dyslexia present difficulties in both verbal and non-verbal tasks, their deficits may be linked to visual factors. On the contrary, if they present difficulties only during the verbal tasks, then the causes of dyslexia may be phonological in nature.

Several studies reported that an abnormal oculomotor pattern is only found during reading tasks (reading short passages vs. a fixation task [14]; reading a text and reading lists of words and of non-words vs. a visual search task [37]; reading pseudowords vs. processing consonant strings [38]; sentence reading vs. dot scanning [39]; and text reading vs. pictogram naming [40]), suggesting that it may be related to a deficit of processing linguistic information. Other studies, however, found an abnormal eye movement pattern during both reading and non-reading tasks (reading isolated words vs. visually guided saccades to LED-targets [41]; text reading vs. visual search [42]; and text reading vs. free exploration of a painting [43]) in line with the idea of an immaturity of cortical structures responsible for visual processing. According to Prado et al. [44] impaired visual attentional processes may contribute to the abnormal eye movements observed in dyslexia.

In order to clarify this issue, Hutzler et al. [38] sought to understand whether abnormal eye movements in German children with dyslexia were associated with a magnocellular deficit or with a phonological deficit at the stage of grapheme-to-phoneme conversion. For this purpose, they compared eye movements in children with and without dyslexia depending on whether they were performing an experimental task involving (i.e., reading pseudowords) or not (i.e., reading consonant strings of different lengths, which is a purely perceptive activity) phonological processing. The authors found more frequent and longer first fixation durations and gaze durations (i.e., the total time spent on each item during first-pass reading) only when the participants with dyslexia read pseudowords. This result suggests that the abnormal oculomotor pattern may be mainly related to a deficit in phonological processing involving grapheme-to-phoneme correspondence (GPC). This finding lends support to the hypothesis that the difficulties reported in readers with dyslexia could be due to linguistic disturbances rather than to perceptual ones. Similarly, Hatzidaki et al. [37] reported abnormal eye movements (more numerous fixations, longer fixation durations, more numerous pro- and retro-saccades) during text reading but not during visual search in Greek children with dyslexia as compared to children without dyslexia. These converging data strengthen the conclusion that difficulties in readers with dyslexia could be mainly related to linguistic information processing and not just to purely visual information processing. All these findings suggest that the abnormal eye movement patterns could be the consequence, rather than the cause, of a phonological deficit. In other words, abnormal eye movement patterns in readers with dyslexia could be due to difficulties occurring during language processing (for instance, difficulties in grapheme-phoneme conversion, reduced visual attention span) [45,46,47].

However, it should be mentioned that the majority of the above-mentioned studies [12,13,14,15,16,17,19,20,22,24,25,26,37,39,40,41,42,43,44] and, generally speaking, of studies measuring eye movements in dyslexia, were conducted in children, and that fewer studies focused on adults [16,48,49]. In adults the phonological and reading processing systems have reached maturity, which makes direct comparisons with the dyslexia population more difficult.

With respect to studies on adults with dyslexia, a recent study by Denis-Noël et al. [48] recorded eye movements in French university students with and without dyslexia while reading pairs of phonologically consistent (e.g., cloche, where –oche can only be pronounced /ᴐʃ/ in French) and inconsistent (e.g., clef where –ef can be pronounced /e/ or /ᴈf/) monosyllabic words. The authors found that the students with dyslexia made more fixations compared to skilled readers, independently of the stimulus type. They also found that when reading inconsistent words, where phonological processes are more solicited in comparison to consistent words, the students with dyslexia had longer fixation durations of the second fixation compared to the first fixation. One possible explanation is that conflicting pronunciations may slow down the recognition process of inconsistent words. The authors claimed that the longer fixation duration could reflect delayed activation of phonological information during reading, which is in line with the phonological deficit hypothesis. It can also be explained by differences in lexical access, since inconsistent words require lexical knowledge, whereas consistent words do not.

Given the contradictory conclusions from previous research, our study aims to shed light on the ongoing debate regarding the underlying causes of dyslexia. Specifically, we aimed to clarify whether dyslexia is primarily linked to linguistic and phonological factors or if visuo-attentional aspects play a more prominent role. We investigated this by examining eye movement evidence in adults with dyslexia during a phonological lexical decision task that used different types of stimuli.

In this study, we compared the eye movements of French university students with and without dyslexia while processing different types of visual stimuli involving either a lexical entry and a grapheme-to-phoneme conversion of varying depth (words < pseudohomophones < pseudowords) or purely visual processing (such as consonant and symbol strings). The participants performed a phonological lexical decision task which consisted in deciding whether each visual sequence presented sounded like a French word. It is worth noting that the task employed in this study required the participants to decide whether the stimulus sounded like a real word, which involves lexical access (in the case of words and pseudohomophones). The strength of the experimental design we used is that it allowed us to test the two major hypotheses discussed in the literature on the causes of dyslexia, namely, the visuo-attentional deficit hypothesis and the phonological one, using a continuum of stimuli ranging from French words to symbol and consonant strings, pseudowords, and pseudohomophones. Several studies investigated neural correlates underlying developmental dyslexia through a phonological lexical decision task (among either children or adults) during EEG [50] and fMRI registration [51,52]. To our knowledge, this is the first study using the phonological lexical decision task to examine eye movements in dyslexia.

Our driving hypothesis was that if participants with dyslexia have a deficit in phonological processing only, abnormal eye movements (longer fixation durations, more numerous fixations, and larger saccade amplitude) in participants with dyslexia should only be found when reading words, pseudohomophones and pseudowords. On the other hand, if abnormal eye movements are observed in participants with dyslexia also when processing consonant and symbol sequences, we can assume that reading difficulties in participants with dyslexia may be associated to poor visual perception and impaired visual attentional processes.

## 2. Materials and Methods

### 2.1. Participants

We tested fourteen native French university students with dyslexia (six males; mean age = 21.2 ± 0.6 years) and eighteen control subjects (five males; mean age = 21.1 ± 0.5 years). The inclusion criteria for both groups comprised the absence of a history of neurological or psychiatric pathology, no drug usage, normal corrected visual acuity (8/10 in each eye, according to Parinaud’s optometric scale), a normal mean intelligence quotient (IQ, evaluated with WISC-IV, between 80 and 115), and no presence of comorbidity symptoms (ADHD or DCD pathologies). For the participants with dyslexia, the diagnosis of dyslexia was made during childhood (mean age of diagnosis = 7.5 ± 2.1 years) by a specialized therapist. The control participants had no history of spelling or reading difficulties. The exclusion criteria encompassed the presence of any known neurological disorders or comorbidities, visual impairment, or drug use.

Note also that all participants with dyslexia had undergone several years of remediation with a speech therapist (mean = 8.4 ± 3.5 years). University students with dyslexia were selected for the study in order to allow us to investigate whether atypical oculomotor patterns and phonological difficulties persist in adults with dyslexia or whether this population has managed to compensate for certain difficulties. Furthermore, due to the demanding requirements of higher education, university students constitute a cohort with a more balanced exposure to reading.

### 2.2. Screening Tests

As reported in Table 1, reading skills, phonological awareness, visuo-attentional skills, and non-verbal intelligence were evaluated with a battery of standardized tests. To assess the reading abilities, we used the French reading test L’Alouette [53] and we took into consideration accuracy and speed of reading, measured by the number of words correctly read per minute and the reading efficiency score (CTL) [54]. The ECLA 16+ battery test [55] was employed to measure several reading abilities (text reading (Pollueur; ECLA 16+ [55]), regular and irregular word reading and pseudoword reading, phonological skills (initial phoneme deletion, spoonerisms, non-word repetition), and rapid letter naming). A five-consonant global report task [9] was used to assess visuo-attentional skills, and the matrices and the similarities subtest of the Wechsler Adult Intelligence Scale IV (WAIS-IV) [56] was used to assess nonverbal intelligence.

The study was approved by the Institutional human experimentation committee of Lille University, France, and was carried out in accordance with the Declaration of Helsinki (Comité Ethique de l’Université de Lille, N° 2020-441-S87).

All participants gave their written consent to participate in the experiment and were paid EUR 15 per hour for their participation.

### 2.3. Linguistic Material

Five experimental conditions were used for the stimulus type: (1) words (e.g., “chaise” (chair) taken from the French database Lexique 3 [57]; all the words were 5-to-6-letter monosyllabic concrete nouns (mean length of the word stimuli = 5.3 ± 0.5) with a high frequency of occurrence (*M* = 148.1, *SD* = 110.9). Orthographic (*M* = 4.8, *SD* = 3.8) and phonological (*M* = 10.9, *SD* = 6.6) neighbors and number of homographs (*M* = 1.4, *SD* = 0.6) and homophones (*M* = 3.5, *SD* = 1.9) were taken into account when selecting the words; (2) Pseudohomophones (stimuli having a phonological but not an orthographic representation in French, or in other words, non-lexicalized stimuli in French but pronounced in the same way as French words; *chèse same pronunciation as “chaise” (chair); Pseudohomophones were created from the list of words by replacing one grapheme at a time with another grapheme corresponding to the same phoneme, adding or eliminating a double consonant or a silent letter [58]; (3) pseudowords (stimuli that are orthographically and phonologically plausible but have neither a phonological nor an orthographic representation in French. They were created from words by changing one grapheme at a time; *chuse); (4) consonant strings (sequences that are orthographically illegal and phonologically unpronounceable, since they contain no vowels and, therefore, no syllables; *nbvrzc). Consonant strings were matched with words based on their form, with respect to the ascender or the descender graphemes that they contained; (5) symbol strings (non-alphabetic stimuli; §Ȼ¥Đ‡). Symbol strings were matched for the number of characters with words. The 12 symbols used in our study were taken from a previous study by Mahé et al. [59]. This ERP study was focused on visual expertise for print and found that the electrical responses of readers without dyslexia were different in response to these symbols compared to alphabetic stimuli, indicating that these symbols are processed differently compared to alphabetic stimuli. In total, 300 stimuli were used, 60 stimuli in each condition.

### 2.4. Eye Movement Recordings

All participants were tested individually in a soundproof room. They were seated 92 cm from a screen, with a chinrest and a forehead rest. Eye movements were recorded using the Eye-link 1000 eye tracker (Eyelink 1000 Desktop Mount, distributed by SR Research Ltd., Mississauga, ON, Canada). Before each session, nine-point gaze calibration was performed and repeated until the validation error was less than 1° on average and less than 1.5° at the worst point. After the calibration session, a phonological lexical decision task was proposed to the 32 participants (see Section 2.5 below). We recorded only the dominant eye of each participant, since previous studies reported no apparent association between ocular dominance and reading skills [60,61].

### 2.5. Procedure

The participants performed a phonological lexical decision task. Each trial started with a fixation cross flashing in the center of a grey screen for 400 ms. The cross was followed by a grey screen flashing for 150 ms and then by the stimulus, which remained on the screen for 700 ms. After the presentation of the stimulus, a question mark appeared on the screen, and the subjects had to indicate as accurately as possible whether the stimulus that was presented sounded like a real word in French or not, by pressing a yes or a no key on the computer keyboard. We chose the offline version of the phonological lexical decision task; the next trial appeared on the screen after the participant’s response. The stimuli were presented in “Arial Narrow” black font, with 47-point lower case letters in the center of the monitor, on a grey background. All 300 stimuli were distributed in equal numbers in 5 blocks, each of which contained 60 trials. The stimuli in each block were pseudorandomized based on the following constraints: no more than two stimuli of the same condition were presented successively; and no more than three stimuli requiring the same response were displayed in succession. Words and their corresponding pseudohomophones were not presented within the same block; the 12 corresponding pseudohomophones of each word within a block were distributed among the four remaining blocks. Eye movements were recorded during the phonological lexical decision task, and calibration was repeated at the beginning of each block presentation.

### 2.6. Data Analysis

During the performance of the phonological decision task by the participants, we measured the mean number of fixations, the duration of the first fixation, and the saccade amplitude, when the stimulus was not processed in a single fixation.

Eye movement analyses were performed using the Data Viewer software (version 4.2.1; SR Research Ltd.). We analyzed eye movements solely during the period of the stimulus appearance on the screen (maximum time 700 ms). We excluded trials of incorrect responses from the data, i.e., when fixations and saccades fell outside the area of interest, and trials including blinks. The percentage of the removed data was 25.5% for the controls and 35.7% for the participants with dyslexia.

### 2.7. Statistical Analysis

The Student’s *t*-test was used to compare reading and cognitive skills in participants with and without dyslexia. A repeated-measures ANOVA was run to compare the response accuracy in the phonological lexical decision task in the two groups of participants.

With respect to the eye movements’ analysis, repeated-measures ANOVAs were conducted for each dependent oculomotor parameter (mean number of fixations, first fixation duration, and saccade amplitude) between the different stimuli (5 levels: word, pseudohomophone, pseudoword, consonant string, symbol string) as a within-subjects factor and between the two groups of participants (dyslexic, control) as a between-subjects factor.

Post-hoc pairwise comparisons were made using a modified Holm procedure. The threshold of statistical significance was set at *p* < 0.05. All statistical analyses were processed using JASP software (a free open-source program for statistical analysis supported by the University of Amsterdam, version 0.16.3.0).

We conducted a power analysis using G*Power 3.1 software [62] to determine the required sample size for our repeated-measures ANOVA with a within -between interaction. The power analysis was conducted a posteriori to ensure that our study had sufficient statistical power to detect meaningful effects. The significance level (alpha) was set at 0.05, and the desired statistical power (1—beta) at 0.80.

## 3. Results

### 3.1. Behavioral Results: Accuracy

Repeated-measures ANOVAs revealed a main effect of the condition applied [*F*(4,120) = 11.01, *MSE* = 146.38, *p* = 0.001, *η*^2^*p* = 0.268]. Post hoc analyses demonstrated fewer correct responses in the pseudoword condition (89.9 ± 13.8) as compared to the word (98.9 ± 1.7%), consonant (98.5 ± 3.9%), and symbol conditions (99.7 ± 0.8%) (*p_holm_* < 0.001, respectively). Given that in our task 120 trials implicated the YES response and 180 trials the NO response, we ran additional *t*-tests on the control group concerning the percentage of error responses in order to exclude that a potential bias in participants’ responses due to the unequal number of YES and NO responses. The percentage of error for words and pseudohomophones was 2.9%, while it was 1.4% for pseudowords, consonant strings, and symbol strings. A paired-samples *t*-test on the control group showed that there was no significant percentage difference between the YES and NO conditions (*p* > 0.05).

**Figure 1:**
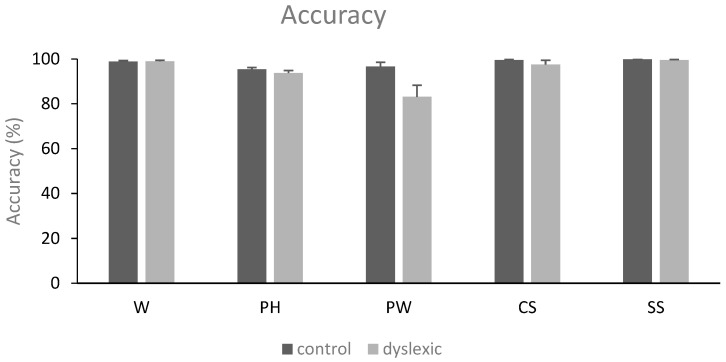
Mean accuracy (in percentage) and standard error of the mean (SEM) for words (W), pseudohomophones (PH), pseudowords (PW), consonant strings (CS), and symbol strings (SS) in the two groups of subjects (controls and dyslexics).

There was also a main effect of the group [*F*(1,30) = 10.48, *MSE* = 45.18, *p* = 0.003, *η*^2^*p* = 0.259], showing that the participants with dyslexia (94.5 ± 6.7%) were less accurate as compared to the control participants (98 ± 2.4%). Lastly, a condition by group interaction was found [*F*(4,120) = 5.22, *MSE* = 146.38, *p* = 0.019, *η*^2^*p* = 0.148]. Post-hoc analyses revealed that only the participants with dyslexia had fewer correct responses in the pseudoword condition compared to all the other conditions (compared to word: *p_holm_* < 0.001; to pseudohomophone: *p_holm_* = 0.004; to consonant: *p_holm_* < 0.001; and to symbol: *p_holm_* < 0.001). See also Figure 1. 

### 3.2. Mean Number of Fixations

**Figure 2:**
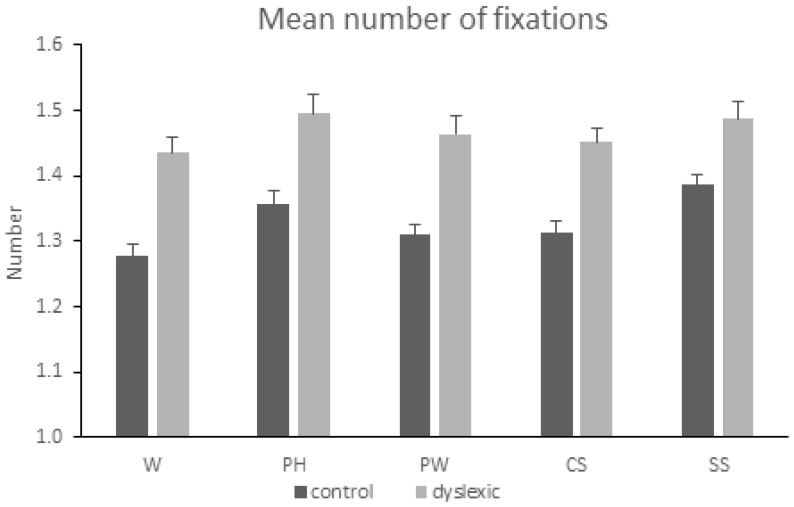
Mean number of fixations and the standard error of the mean (SEM) when reading words (W), pseudohomophones (PH), pseudowords (PW), consonant strings (CS), and symbol strings (SS) in the two groups of subjects (controls, dyslexics).

ANOVA showed a main effect of the condition [*F*(4,472) = 4.784, *MSE* = 0.029, *p* = 0.001, *η*^2^*p* = 0.039]. Post -hoc analyses revealed more fixations in the pseudohomophone and the symbol condition compared to the word condition (*p_holm_* = 0.011 and *p_holm_* = 0.002 respectively). In addition, a significant main effect of the group [*F*(1,118) = 67.415, *MSE* = 0.043, *p* < 0.001, *η*^2^*p* = 0.364] was found, reflecting more fixations by the group of participants with dyslexia as compared to the control group. The condition-by-group interaction was not significant [*F* < 1]. See also Figure 2.

### 3.3. First Fixation Duration (in Trials with Double Fixations)

**Figure 3:**
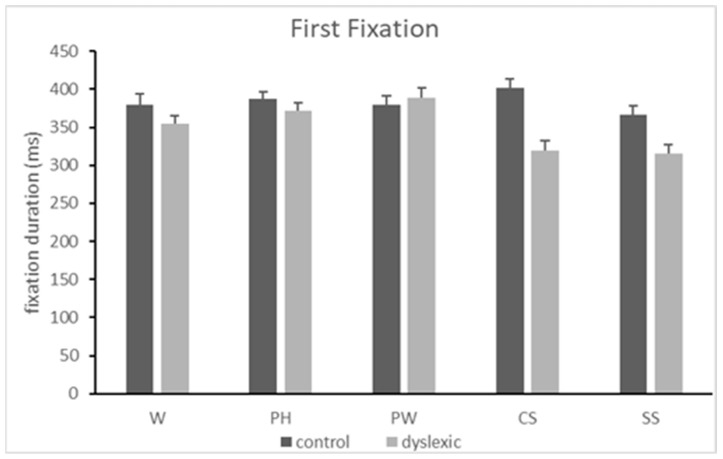
Mean duration of the first fixation (in ms) and standard error of the mean (SEM) when reading words (W), pseudohomophones (PH), pseudowords (PW), consonant strings (CS), and symbol strings (SS) in the two groups of subjects (controls and dyslexics).

There was a main effect of the condition [*F*(4,448) = 4.206, *MSE* = 7855.491, *p* = 0.002, *η*^2^*p* = 0.039]. Post -hoc analyses revealed that the first fixation was shorter for symbols compared to pseudohomophones (*p_holm_* = 0.011) and pseudowords (*p_holm_* = 0.003). There was also a main effect of the group [*F*(1,112) = 18.313, *MSE* = 8320.018, *p* < 0.001, *η*^2^*p* = 0.141], indicating that the control participants had longer first fixation durations compared to the participants with dyslexia (mean difference = 32.699, ms). Lastly, there was a condition-by-group interaction [*F*(1,448) = 4.206, *MSE* = 7855.491, *p* = 0.002, *η*^2^*p* = 0.039]. Post hoc analyses revealed that it was only in the group of participants with dyslexia that the first fixation durations were longer for pseudohomophones and pseudowords compared to symbols (*p_holm_* = 0.028 and *p_holm_* < 0.001 respectively). They were also longer for pseudowords compared to consonant strings (*p_holm_* = 0.001) only for the group of participants with dyslexia. See also Figure 3.

### 3.4. Saccade Amplitude

**Figure 4:**
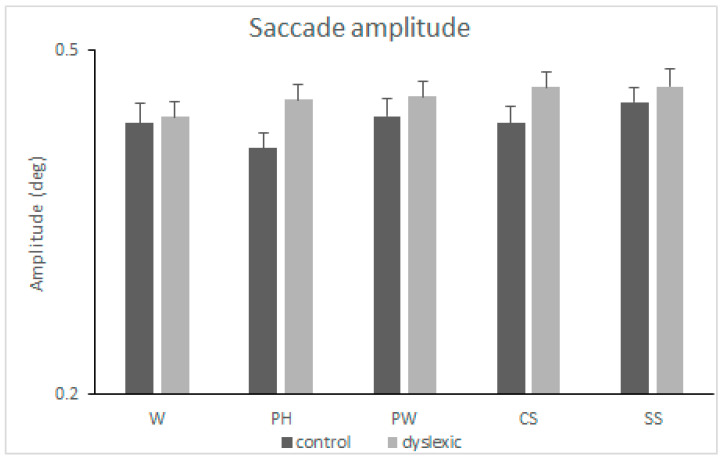
Mean amplitude of saccades (degrees) and standard error of the mean (SEM) measured when reading words (W), pseudohomophones (PH), pseudowords (PW), consonant strings (CS), and symbol strings (SS) in the two groups of subjects (controls and dyslexics).

ANOVA revealed a main effect of the group [*F*(1,112) = 4.769, *MSE* = 0.014, *p* = 0.031, *η*^2^*p* = 0.041], with the dyslexia group having longer saccade amplitudes compared to the control group (mean difference = 0.022). Neither the main effect of the condition [*F*(4,448) = 1.189, *MSE* = 0.011, *p* < 0.315] nor the condition-by-group interaction [*F* < 1] were found to be significant. See also Figure 4.

With respect to the power calculation, in the analysis of the within -between interaction effects using a repeated-measures ANOVA, we obtained a range of results, some of which indicated that our total sample size of 32 participants was sufficient to detect significant effects, while others raised concerns about power limitations.

## 4. Discussion

To the best of our knowledge, this is the first study comparing oculomotor patterns in students with and without dyslexia during a phonological lexical decision task. Our behavioral results indicated that the Participants with dyslexia exhibited lower performance when reading pseudowords in comparison to the control group. Regarding eye movements, the most important findings are as follows: (1) the participants with dyslexia made more fixations compared to the control participants, independently of the stimulus type; (2) the participants with dyslexia performed longer first fixations for pseudowords and pseudohomophones compared to symbols; (3) the control participants reported longer first fixation durations and shorter saccade amplitudes compared to the participants with dyslexia. Lastly, concerning the stimulus type, we found fewer fixations for words compared to pseudohomophones and symbols. These findings are discussed below.

The main finding of the study is that the participants with dyslexia exhibited a higher number of fixations than the control participants, regardless of the type of stimulus. These findings are in accordance with the visuo-attentional deficit in dyslexia suggested by Bosse et al. [9]. The lower number of letters processed in parallel and the shorter visual attentional span (as shown in Table 1) could lead to the abnormal oculomotor pattern reported in the participants with dyslexia during reading, in line also with the findings reported in children with dyslexia during a reading task as well as during a visual search task [19]. In addition, the absence of a condition-by-group interaction highlights difficulties in all stimuli independently of their linguistic information. This result is in accordance with studies finding deficiencies associated with both linguistic and non-linguistic stimuli, supporting the idea that visuo-attentional and not phonological deficiencies could better explain reading problems in people with dyslexia [31].

However, we cannot exclude the existence of a phonological deficit. Our behavioral observations align with prior research [59] by indicating that the individuals with dyslexia exhibited lower accuracy compared to the control readers, notably, when reading pseudowords, although our stimuli were short. These results provide evidence for the hypothesis that individuals with dyslexia encounter challenges when reading pseudowords, which have neither orthographic nor phonological representations, and this, thereby, places additional strain on the grapheme-to-phoneme conversion process. In addition, screening tests of dyslexia indicated a significant difference between participants with and without dyslexia in tests measuring phonological awareness (i.e., initial phoneme deletion, spoonerisms; see also Table 1). The fact that participants with dyslexia make more fixations may indicate that their reading abilities are deficient and that they preferentially use the sublexical grapheme-to-phoneme conversion procedure, whatever the type of stimulus to be read [48,49]. Excessive fixations during word reading often stem from a dominant sublexical reading strategy [25]. Hawelka et al. [49] tried to connect the dual-route model [63] and the E-Z Reader model [64] in dyslexia, by explaining that the high number of fixations and saccades observed in readers with dyslexia signifies difficulties in the lexical route and in orthographic whole-word recognition, ultimately leading to a reliance on sublexical processing. More precisely, they support the dual-route model, which posits the use of two separate routes, namely, the lexical and the sublexical routes, by proficient readers during word recognition. Within the context of dyslexia, readers with a deficient orthographic lexicon rely more on sublexical processing when they cannot find a matching entry in their deficient orthographic lexicon, even for relatively frequent and short words, as was the case in this study. In parallel, the E-Z Reader model, which focuses on eye movement and attention allocation during reading, suggests that a failure in the “familiarity check” may indicate a deficiency in the orthographic lexicon, indicating that the reader cannot rapidly recognize whole words.

Similar results were found in children with dyslexia by De Luca et al. [25], who reported more frequent fixations while reading short pseudowords and longer stimuli (words and pseudowords) in participants with dyslexia compared to control participants, and by Hutzler et al. [24], who found a higher number of fixations in children with dyslexia compared to children without dyslexia when reading pseudowords.

The absence of a group-by-condition interaction with stimuli requiring a grapheme-to-phoneme conversion cannot lead to a straightforward indication in favor of the phonological deficit but may indicate that the different eye movement patterns found in people with dyslexia may be attributed to both phonological and visuo-attentional deficits. Furthermore, the observation that the participants with dyslexia made more fixations irrespective of whether the stimulus contained lexical or just sublexical information, strengthens the hypothesis of the presence of both phonological and visuo-attentional difficulties in dyslexia. Unfortunately, we are not able to discriminate whether this abnormal oculomotor pattern is the origin or the cause of dyslexia [65]. This could be highlighted by an interaction with the type of stimulus, which could better indicate whether a deficit has a phonological or a visuo-attentional cause. We also note here the study by Castet et al. [65] which supports the idea that iconic memory and short-term visual memory play an important role in the visuo-attentional deficits found in dyslexia. These deficits may coexist with phonological deficits, leading to a multielement deficit model of dyslexia.

Another result indicating differences between the participants with and without dyslexia is that first fixation durations were found to be longer in the participants with dyslexia when reading pseudohomophones and pseudowords compared to symbol stimuli. This result may indicate that the phonological code is not readily activated during the early processing stages of visual word recognition, since a prolonged first fixation duration was observed for stimuli requiring phonological processing, i.e., pseudohomophones and pseudowords. This can be associated with the phonological deficits found in readers with dyslexia. On top of that, the fact that the first fixation durations are longer for phonologically challenging stimuli (pseudohomophones and pseudowords) compared to symbols that require solely visual processing supports the idea that phonological deficits are more prominent in dyslexia compared to visual ones. Combining this finding with the previous one regarding more numerous fixations and reducing it to the dual-route model and the E-Z Reader model, we can infer that individuals with dyslexia not only may rely on a sublexical processing system but also may present a delayed activation of the phonological code. These results are in accordance with studies supporting deficiencies only with linguistic stimuli but not with symbols [33,34,36].

In the study by Denis-Noël et al. [48], students with dyslexia had longer second fixation durations during the reading of inconsistent words, for which phonological processing is more demanding. However, in our study, we found longer first fixation durations as in Denis-Noël et al. when the dyslexic participants were engaged in reading phonologically challenging stimuli (pseudohomophoness and pseudowords). This difference can be attributed to the fact that we used pseudohomophones and pseudowords, which are not lexicalized stimuli, to examine the phonological processing during reading rather than consistent and inconsistent words; our word stimuli were therefore short, high-frequency concrete words.

However, we note that this result cannot rule out the possibility that visuo-attentional deficits are also present in dyslexia, since, on top of the finding concerning more numerous fixations independently of the condition in dyslexia, the global visuo-attentional span showed a clear difference between the two groups (see Table 1). The group difference found with respect to the first fixation duration strengthens this interpretation. More precisely, the control participants performed longer first fixation durations as compared to the participants with dyslexia. This indicates that individuals without dyslexia potentially process a greater portion of the stimulus during their initial fixation.

Another result in favor of visual deficits in dyslexia concerns the saccade amplitudes. Surprisingly, contrary to previous research indicating shorter saccade amplitudes in individuals with dyslexia, our study showed that the control participants reported shorter saccade amplitudes compared to the participants with dyslexia. This unexpected and contradictory result can be attributed to the specific nature of the task employed, which was not a typical reading task for the examination of eye movements. In the present task, isolated short stimuli were presented on the screen rather than in a list of words or in a text as in previous studies on dyslexia. We interpret this result as an indication that the control group exhibited a higher level of concentration on the visual area of interest. They processed the stimuli effectively within a single fixation, and maintained their attention without losing focus. As a result, their saccade amplitudes were shorter. In the present context, larger saccade amplitudes can be understood as deviation from the intended fixation point. Given that the control group displayed less deviation with respect to the fixation visual area of interest, we can infer that they possessed better attentional abilities compared to the participants with dyslexia. This interpretation could support the theory that individuals with dyslexia may have poorer visuospatial abilities [66] and experience visual impairments, indicating that they often fixate on incorrect locations [43,67,68]. More ecological reading situations could better illustrate how individuals with dyslexia navigate visual tasks, shedding further light on their visuospatial difficulties and fixation tendencies.

Furthermore, the observed decrease in saccade amplitude among the control participants could also suggest a potentially larger visual span compared to the participants with dyslexia, given the relative brevity of the stimuli, typically consisting of 5-6 letters or symbols. In other words, if the stimulus was not processed in one fixation, processing the remainder of the stimulus outside of the visual span required a smaller adjustment of the fixation point in the control participants compared to the participants with dyslexia.

In our study, we found fewer fixations in the word condition compared to the pseudohomophone and symbol conditions. This result, which is in accordance with previous studies [25,49], can be partially explained by the fact that reading words is acquired through the direct route of reading [63], whereas reading pseudohomophones, which are stimuli with no orthographic representation, needs the application of grapheme-to-phoneme conversion rules. In the case of the comparison between word and symbol, the less numerous fixations reported for symbols could be due to the fact that this type of stimulus was unfamiliar and required more fixations to be processed compared to a familiar stimulus such as words. Additionally, when it comes to processing symbols, individuals generally find it to be a less familiar task compared to processing alphanumeric stimuli. The visual processing of symbol stimuli tended to be more effortful for all participants involved. They demonstrated a decreased likelihood of processing the entire string in one fixation, especially in comparison to all other conditions (except pseudohomophones), and exhibited shorter first fixation durations. These findings suggest a difficulty in encompassing the entire symbol string within their visual span, unlike other types of stimuli. Furthermore, the higher number of fixations observed in response to pseudohomophones, as opposed to words, can be attributed to the demands of the phonological lexical decision task. Processing pseudohomophones requires the greatest amount of phonological processing to yield a correct answer. Word stimuli, on the other hand, may be processed as a singular unit in certain trials. Similarly, consonant strings are easily dismissed as unpronounceable, while pseudowords can be eliminated once they deviate noticeably from any known word pronunciation. Only pseudohomophones must be fully processed in order to provide a correct answer. These differences in phonological processing difficulty were more prominent in the participants with dyslexia.

Surprisingly, our data did not show any significant group difference in terms of the number of fixations when reading stimuli that required a grapheme-to-phoneme conversion (pseudowords and pseudohomophones). A similar result was reported by De Luca et al. [25] in children when short words and pseudowords were presented. In the present study, this can most likely be attributed to the fact that the stimuli used were short and high-frequency concrete nouns and/or to the fact that university students with dyslexia could have developed some compensatory mechanisms to enhance their reading performances (in addition to benefiting from their training sessions with speech therapists).

### Limitations

The short and frequent monosyllabic stimuli used in this study together with the fact that the subjects were university students who had completed several years of remediation and had acquired strategies to compensate for their reading difficulties may be the cause of the similarities in the oculomotor pattern observed between participants with and without dyslexia. In addition, our experimental design may have been too simple, since the stimuli were presented alone in the center of the screen after the presentation of a center fixation cross; consequently, the subject was already fixating the center of the screen. Future research in a more ecological situation of reading a text and with a larger population is needed in order to explore the oculomotor patterns in students with dyslexia as a function of the amount of remediation.

## 5. Conclusions

To sum up, our study focused on comparing eye movements in French adults with and without dyslexia during a phonological lexical decision task in order to better distinguish the role of phonological and/or visuo-attentional deficits in dyslexia. More numerous fixations in the subjects with dyslexia confirm a deficit in their decoding abilities and a less automatic processing during reading. They also support the existence of a visuo-attentional deficit, since more numerous fixations were found independently of the stimulus. At the same time, the reduced visuo-attentional span reported in the participants with dyslexia could be the cause of their need to make more fixations in order to process stimuli. Furthermore, longer first fixation durations during processing phonologically challenging stimuli (pseudohomophones and pseudowords) compared to stimuli requiring solely visual processing (consonants and symbols) support the existence of a delayed phonological processing and thus support the presence of a phonological deficit in dyslexia. Taken together, the present results support the coexistence of a phonological and a visuo-attentional deficit in dyslexia. However, it still remains an open question whether these abnormal eye movements are one of the causes or one of the consequences of dyslexia. We believe that phonological together with visuo-attentional remediation could be useful to improve the phonological and visuo-attentional span performances in participants with dyslexia [66], as for example in the study by Harrar-Eskinazi et al. [69], who focused on the multifactorial nature of dyslexia in order to examine the potential benefits of multimodal interventions.

## Figures and Tables

**Table 1:** Assessment of reading and other cognitive functions in the participants. Mean value (±standard error of the mean) for the different tests run in the two groups of participants (control readers and dyslexic readers), *p* values derived from student’s *t*-tests are also reported.

	Controls *N* = 18	Dyslexics *N* = 14	*p* Values
Age (years)	21.1 ± 0.5	21.2 ± 0.6	0.895
Meaningless text reading (words correctly read/minute)	143 ± 5.9	102 ± 4.3	<0.001
Meaningless text reading (reading efficiency score, CTL)	430 ± 18	305 ± 13	<0.001
Text reading (words correctly read/minute)	205 ± 5.8	148 ± 7.8	<0.001
Regular word reading (score/20)	19.3 ± 0.2	18.7 ± 0.3	0.066
Regular word reading (time in seconds)	11.0 ± 0.6	19.9 ± 1.9	<0.001
Irregular word reading (score/20)	18.8 ± 0.4	17.6 ± 0.5	0.055
Irregular word reading (time in seconds)	10.6 ± 0.6	18.1 ± 1.7	<0.001
Pseudoword reading (score/20)	18.6 ± 0.3	17.0 ± 0.6	0.015
Pseudoword reading (time in seconds)	16.2 ± 1.4	33.2 ± 2.5	<0.001
Initial phoneme deletion (score/10)	8.9 ± 0.3	7.9 ± 0.7	0.151
Initial phoneme deletion (time in seconds)	38.8 ± 2.8	50.3 ± 2.4	0.005
Spoonerisms (score/20)	18.7 ± 0.3	14.1 ± 1.2	<0.001
Spoonerisms (time in seconds)	89.2 ± 8.5	216 ± 31	<0.001
Non-word repetition (score/20)	19.0 ± 0.1	18.6 ± 0.3	0.220
Non-word repetition (time in seconds)	68.8 ± 2.2	78.6 ± 3.2	0.014
Rapid automatized naming (RAN) letter (score/50)	49.6 ± 0.1	47.7 ± 1.7	0.211
Rapid automatized naming (RAN) letter (time in seconds)	16.1 ± 0.7	22.4 ± 1.5	<0.001
Visuo-attentional span (score/100)	93.1 ± 1.4	75.9 ± 4.2	<0.001
Similarities subtest WAIS IV	10.8 ± 0.5	10.1 ± 0.6	0.390
Matrices subtest WAIS IV	10.5 ± 0.6	10.1 ± 0.3	0.614

## Data Availability

The datasets generated and/or analyzed during the current study are available from the corresponding author on reasonable request.
